# Characterization of a transgender population in Portugal and the portuguese outlook on transgender health care

**DOI:** 10.1192/j.eurpsy.2025.779

**Published:** 2025-08-26

**Authors:** C. P. Desport, I. G. Carvalho, Z. Figueiredo, S. Viveiros

**Affiliations:** 1 Mental Health, Unidade Local de Saúde de Santo António, Oporto, Portugal

## Abstract

**Introduction:**

Gender incongruence (GI) and gender dysphoria (GD) represent a health condition in which the individual’s gender identity does not correspond to their assigned gender at birth, when dysphoria is present it means there is significant distress. This population needs specific healthcare from a multidisciplinary team involving psychiatry, psychology, endocrinology, urology and/or gynecology, otorhinolaryngology and plastic surgery. In Santo Antonio Hospital, currently, Unidade Local de Saúde de Santo António (ULSSA), there is a sex and gender unit (USEG – Unidade de Sexo e Género), with the previous described specialties, that evaluates and follows transgender individuals through their transition process. A mental health assessment is a major part of this process since, in Portugal, it is required an evaluation, from a mental health professional with clinical experience in this field, to have hormonal treatment, and two to have surgical procedures done.

**Objectives:**

Characterization of a population of transgender individuals in Portugal, framing the results in the current Portuguese panorama of transgender health care.

**Methods:**

Retrospective study of individuals that attended sexology/psychiatry consultation from USEG. Age, type of treatments they have made so far and expectations, type of transition – male to female (MtF) or female to male (FtM) or non-binary (NB), occupation and education, relationship status, city of origin and comorbidities were analyzed.

**Results:**

143 people were assessed and/or followed for gender incongruence and/or dysphoria, with a minimum age of 17 and a maximum of 61 at the time of their first consultation, mean age of 24,4 years. Of these 49,65% had FtM GI/GD, 38,46 % MtF and 11,89% identified as NB. Several individuals had concomitant medical conditions, and there were high rates of psychiatric comorbidity like anxiety and depression but also neurodevelopmental disorders. The majority of the individuals intended to start hormonal treatment with many expressing fear and anxiety related to the surgical procedures but with a still high proportion looking forward to them in the future. Many of our patients lived in places far from our hospital.

**Conclusions:**

We found a higher prevalence of FtM than MtF, which is in contrast with most studies in the field but similar to another Portuguese study. The high percentage of medical, specifically psychiatric comorbidities, enhances the importance of a mental health assessment and follow up in this population. A significant percentage of our patients came from cities far away reflecting the scarcity of specialized trans health care in our country. GD and GI diagnosis is increasing worldwide and the transition process is long and highly complex requiring a multidisciplinary team that can collaborate on a unique individual’s care in a coordinated and safe way.

**Disclosure of Interest:**

None Declared

